# Editorial: Molecular mechanisms of metal toxicity and transcriptional/post-transcriptional regulation in plant model systems

**DOI:** 10.3389/fpls.2024.1502021

**Published:** 2024-11-27

**Authors:** Debasis Chakrabarty

**Affiliations:** Molecular Biology and Biotechnology, National Botanical Research Institute (CSIR), Lucknow, Uttar Pradesh, India

**Keywords:** heavy metal-, gene regulation, genomics, epigenetic changes, small RNA (miRNA)

## Introduction

The intricate relationship between plants and their environment has become the focus of research in recent years, particularly at the molecular and physiological levels. Plants, being sessile organisms, are constantly exposed to a range of environmental stressors, including toxic compounds like heavy metals (HMs). These HMs can significantly disrupt various physiological and metabolic processes in plants, negatively impacting their growth, development, and productivity. There are several studies on the multilevel molecular mechanisms involved in plant responses to abiotic stress, but limited research focused specifically on responses to HM exposure. Several researchers have identified significant molecular differences in plant responses to HM stress (Xie et al., 2023; Gao et al., 2023). However, the specific molecular mechanisms by which heavy metals affect plants remain underexplored. Thus, there is a critical need to integrate omic approaches—such as proteomics, transcriptomics, and genomics—to improve our understanding of the physiological and molecular mechanisms underlying HM responses in crops (Dubey et al., 2023; Tiwari et al., 2022a). This includes summarizing key transcription factors (TFs) (Dutta et al., 2024), proteins, and miRNAs (Dubey et al., 2021) involved in HM tolerance and exploring their potential for developing resistant crop varieties. Various genes and TFs have also been identified to play a role in metal stress adaptation. GSTU5 improves arsenic (As) tolerance in rice by chelating As in the root vacuole and limiting its translocation to the shoot (Tiwari et al., 2022b). Kidwai et al. (2019) reported that *OsPRX38* enhances plant tolerance to As stress by reducing As accumulation via lignin biosynthesis and activating the signaling network of different antioxidant systems. OsGrx_C7, upregulated by arsenite (AsIII), enhanced rice tolerance by reducing grain As accumulation via regulation of root growth and AsIII transport through changes in aquaporin expression (Verma et al., 2020).

miRNAs play a crucial role in plant responses to HM stress by influencing gene expression at both the transcriptional and post-translational levels (Singh et al., 2021). They are known to play a central role in the transcriptional regulation of gene networks in response to metal stress. This is evident as many target genes of miRNAs encode transcription factors and proteins involved in metabolic processes and responses to metal stress. Different plant varieties exhibit different miRNA expression patterns in response to exposure to various HMs, such as cadmium (Cd), chromium (Cr), As, lead (Pb), mercury (Hg), and aluminum (Al) (Dubey et al., 2021). For example, a study on cadmium stress reported that the miR166-OsHB4 module regulates Cd accumulation and tolerance in rice. miR166 expression is downregulated in rice under Cd stress. Conversely, overexpression of miR166 enhances Cd tolerance, mitigates oxidative stress, and reduces Cd translocation from roots to shoots and accumulation in rice grains. Altered expression levels of the primary target of miR166, OsHB4, exhibit corresponding changes in Cd tolerance in rice plants (Ding et al., 2018). In addition to miRNAs, HM stress also activates various signaling pathways involving reactive oxygen species (ROS), nitric oxide (NO), hormones like auxin, cytokinin, and ethylene, along with calcium and MAPK signaling (Tang et al., 2023; Singh et al., 2021). Abscisic acid (ABA) mediates the activation of the MAPK signaling pathway in response to Cd toxicity. The BvPYL9 protein serves as a key regulator within this cascade, contributing to the hormetic effects of low-level Cd exposure on sugar beet (Zhao et al., 2024). NO was discovered to regulate metal transporters, especially ABC, NIP, NRAMP and iron transporters, in addition to stress-related genes such as GSTs, CytP450, GRXs, TFs, signaling, amino acids, hormone(s) and secondary metabolism genes involved in As detoxification (Singh et al., 2017). Cd-induced ABA upregulates ABSCISIC ACID-INSENSITIVE5 (ABI5), a basic region/Leu zipper transcription factor that interacts with the R2R3-type MYB transcription factor, MYB49. This prevents the binding of MYB49 to the promoter of the downstream genes (*bHLH38*, *bHLH101, HIPP22* and *HIPP44*) involved in Cd uptake, thus reducing Cd accumulation (Zhang et al., 2019).

Recent research highlighted epigenetic marks, particularly DNA methylation, as crucial regulators of abiotic stress-related gene expression that influence the activity of stress-responsive genes. DNA methylation patterns are consistently associated with HM exposure in plants, indicating that methylation plays a dual role in HM stress responses (Aina et al., 2004; Choi and Sano, 2007). Evidence suggests that methylation can protect against HM-induced DNA damage and regulate gene expression, with specific changes linked to transcriptional variation and transgenerational inheritance of stress tolerance (Gallo-Franco et al., 2020; Oono et al., 2016; Feng et al., 2016; Cong et al., 2019). Cong et al. (2024) revealed a strong correlation between elevated expression of Hg resistance genes and DNA hypomethylation in the putative promoter regions of these genes in an Hg-resistant rice line (RHg) derived from a heterozygous OsMET1-2 (DNA methyltransferase-coding gene) mutant. This finding indicates an epigenetic basis for mercury resistance in this plant. Reduced methylation levels likely facilitate greater accessibility of these genes to the transcriptional machinery, leading to increased expression and enhanced mercury tolerance. Despite numerous studies, significant research gaps remain in understanding the epigenetic regulation of heavy metal and metalloid stress responses in plants. Further research is needed to clarify the specific mechanisms and effects of epigenetic modifications under such environmental stresses.

## Objectives

Despite progress in identifying key components involved in HM tolerance and elucidating HM toxicity, numerous questions remain unanswered. Furthermore, different HMs can elicit distinct toxicity symptoms, and plants employ different defense strategies to counteract specific metal stressors. This Research Topic aims to address these knowledge gaps by investigating the regulatory mechanisms governing metal toxicity and the underlying molecular processes. By addressing these issues, this Research Topic aims to contribute to a deeper understanding of plant metal stress responses and provide valuable insights for the development of strategies to mitigate the negative impact of heavy metals on plant health and productivity.

One such study investigated the effects of magnesium stress on flavonoid biosynthesis in sweet orange ‘Newhall’ peels. Flavonoids are important secondary metabolites that are considered to have benefits in nutrition, food, and medicine. However, little is known about the molecular mechanisms regulating flavonoid biosynthesis under Mg stress. Scientists performed an integrated metabolome and transcriptome analysis of sweet orange peels under Mg-deficient and Mg-sufficient conditions. Overall, this investigation revealed high variability in flavonoid composition and an increase in the total flavonoid content under Mg deficiency. The current study identified 1,533 secondary metabolites, of which 740 were flavonoids, with flavones constituting their main component. There were 17,897 differentially expressed genes that were enriched and participated in flavonoid pathways by transcriptomic analysis. Weighted Gene Correlation Network Analysis found six structural hub genes and ten transcription factor hub genes related to flavonoid biosynthesis, while *CitCHS* was the key gene controlling flavone synthesis. These results shed light on the metabolism of flavonoids under Mg stress and give more insight into the mechanisms at the molecular level, but they also suggest strategies for the cultivation of high-flavonoid plants (Xiong et al.).

The other important aspect of the research is related to the response of *Ligusticum chuanxiong* to cadmium stress. Cadmium is one of the highly toxic metals known to considerably hamper plant growth and productivity. The researchers pointed out that Cd stress inhibited biomass accumulation and root development but activated the antioxidant system of the plant. Cd accumulates in root tissues, distributing mainly in the soluble fraction and the cell wall. Transcriptome analysis indicated that genes related to photosynthetic pathways were downregulated, while some genes encoding plant hormones and antioxidant systems responded positively to Cd regulation. Many genes known to be involved in cell wall modification were upregulated, which may indicate an improvement in the potential of the root cell wall to sequester more Cd. Key metal transport proteins, such as ATPases, MSR2, and HAM3, were implicated in Cd translocation from the apoplast to the cell membrane, highlighting the important role played by ABC transport proteins in intravesicular compartmentalization and efflux of Cd. These results reveal the minute molecular responses of *L. Chuanxiong* to Cd stress and underline the role of the antioxidant system and cell wall modifications in reducing Cd toxicity (Zhang Z. et al.)

Another critical area of research is the toxicity of chromium (Cr) in plants since Cr is a trace metal harmful to plants and a human carcinogen present in the environment due to industrial and anthropogenic activities. Cr(VI) is more toxic; it interferes with several physiological and metabolic plant pathways by increasing ROS activity. Plants have developed a number of mechanisms for tolerance to Cr toxicity, such as vacuolar absorption and accumulation of Cr, its immobilization with organic chelates, and extraction by various transporters and ion channels. The key proteins for Cr sequestration are metallothioneins, phytochelatins, and glutathione-S-transferases. Several genes and transcription factors, including WRKY and AP2/ERF, take part in the defense against Cr stress. It is now well established that OMICs approaches, including genomics, proteomics, and transcriptomics in metallomics, have contributed to great advances in the knowledge of Cr-stress tolerance in plants. This review exhaustively presents a model of Cr-plant interactions, detailing Cr uptake, translocation, and accumulation in plants, emphasizing the potential of systems biology and integrated OMIC approaches to improve Cr-stress tolerance in plants (Abdullah et al.).

Another study investigated the bioremediation potential of *Gracilaria bailinae* in Cd-contaminated waters, thus making macroalgae efficient tools for bioremediation. In this respect, the absorption and accumulation capacity for Cd by *G. bailinae* were assessed based on physiological and biochemical analyses in the event of Cd exposure coupled with transcriptomic analysis. The study showed that at low Cd concentrations, G. bailinae showed stable growth; however, the higher Cd concentrations had striking impacts on plantlet growth and antioxidant enzyme activities. A large number of DEGs involved in peptidase activity, ion transport, and metabolism were identified through transcriptome analysis. Under low Cd stress, the overexpression of DEGs related to histidine metabolism and the antioxidant pathway significantly promoted cell wall regeneration and enhanced antioxidant enzyme activities. bailinae to Cd stress and underscoring its potential for bioremediation in Cd-contaminated waters (Li et al.).

Another area of interest is the interaction between the plant growth-promoting rhizobacterium *Staphylococcus arlettae* and *Helianthus annuus* under arsenic (As) stress. As stress significantly diminishes the relative growth rate and net assimilation rate of *H. annuus*. In the presence of *S. arlettae*, which exhibits tolerance to As, there is enhanced plant growth in As-contaminated media. *S. Arlettae* helps to transform As into more accessible forms for plants, increasing its uptake and accumulation. The bacterium enhances plant enzymatic antioxidant systems that include superoxide dismutase, peroxidase, ascorbate peroxidase, catalase, and also non-enzymatic antioxidants such as flavonoids and phenolics, glutathione. Moreover, *S. arlettae* induces the production of osmolytes like proline and sugars, which mitigate water loss and maintain cellular osmotic balance under As-induced stress. Malonaldehyde content is also reduced, and electrolyte leakage is minimized by the bacterium, counteracting the As toxicity. The findings underscore that *S. arlettae* may have the potential to mitigate plant growth against As toxicity and favor plant growth in contaminated environments (Qadir et al.).

Another study in potato (*Solanum tuberosum*) addressed phosphorus deficiency and aluminum toxicity in acidic soils, which act as significant constraints on crop yield. In this study, members of the potato *StPHR* gene family were identified, of which StPHR1 is the key regulator responding to phosphorus deficiency and aluminum toxicity. Further bioinformatic analysis revealed that the expression level of *StPHR1* was highly induced in potato roots under stress. Subcellular localization, GUS staining, heterologous overexpression, and protein interaction studies experimentally confirmed StPHR1’s regulatory function in the nucleus to mediate resistance to both stresses. Heterologous expression of StPHR1 in *Arabidopsis* resulted in a growth phenotype resistant to aluminum toxicity and phosphorus deficiency with reduced Al content in roots. It also identified an interaction between StPHR1 and StALMT6 for applications in improving potato resistance against nutrient deficiency and toxic metal stress in acidic soils (Zhang F. et al.).

Taken together, these studies further our current understanding of the molecular mechanisms underlying metal toxicity and transcriptional/posttranscriptional regulation in plant model systems. They illustrate the intricate interplay of metabolic pathways, gene expression, and stress responses that can be used in the design of strategies to improve plant resilience and use in environmental bioremediation. It would allow the identification of regulatory networks and key mechanisms that control plant responses to metal stress, the modulation of which may mitigate the adverse effects of metal toxicity and thus help in sustainable agriculture and the environment.

## Future research areas

Future research on plant responses to metal stress should prioritize several key areas to improve our understanding along with agricultural outcomes. First, the integration of multi-omic approaches—combining genomics, transcriptomics, proteomics, and metabolomics—can provide a comprehensive view of the complex biological responses to metal exposure. This holistic perspective is essential for identifying critical pathways and molecular mechanisms involved in metal stress responses. Second, elucidating epigenetic mechanisms is crucial, as these processes can regulate gene expression without altering the underlying DNA sequence. Understanding how epigenetic modifications, such as DNA methylation and histone modifications, influence plant responses to metal stress may reveal new strategies for enhancing tolerance. Exploring plant-microbe interactions also holds promise, as beneficial microbes can play a significant role in mitigating metal toxicity and enhancing plant resilience. Research in this area can lead to innovative biotechnology applications that leverage these interactions for crop improvement. Furthermore, developing targeted crop improvement strategies, including breeding programs that incorporate tolerance traits identified through omics and epigenetic studies, can help create varieties that are better suited for metal-contaminated soils. Finally, addressing the implications of metal toxicity for human health is critical, as heavy metals can accumulate in the food chain, posing risks to consumers. By focusing on these areas, we can deepen our understanding of plant metal stress responses, improve crop tolerance, and mitigate the negative impacts of heavy metals on both agricultural productivity and human health.
